# A Voxel-Based Morphometry Study Reveals Local Brain Structural Alterations Associated with Ambient Fine Particles in Older Women

**DOI:** 10.3389/fnhum.2016.00495

**Published:** 2016-10-13

**Authors:** Ramon Casanova, Xinhui Wang, Jeanette Reyes, Yasuyuki Akita, Marc L. Serre, William Vizuete, Helena C. Chui, Ira Driscoll, Susan M. Resnick, Mark A. Espeland, Jiu-Chiuan Chen

**Affiliations:** ^1^Department of Biostatistical Sciences, Wake Forest School of MedicineWinston-Salem, NC, USA; ^2^Department of Preventive Medicine, University of Southern CaliforniaLos Angeles, CA, USA; ^3^University of North CarolinaChapel Hill, NC, USA; ^4^Department of Neurology, University of Southern CaliforniaLos Angeles, CA, USA; ^5^Department of Psychology, University of Wisconsin-MilwaukeeMilwaukee, WI, USA; ^6^Laboratory of Behavioral Neuroscience, Intramural Research Program, National Institute on Aging, National Institutes of HealthBaltimore, MD, USA

**Keywords:** air pollution, brain, MRI, PM2.5, VBM

## Abstract

**Objective:** Exposure to ambient fine particulate matter (PM_2.5_: PM with aerodynamic diameters < 2.5 μm) has been linked with cognitive deficits in older adults. Using fine-grained voxel-wise analyses, we examined whether PM_2.5_ exposure also affects brain structure.

**Methods:** Brain MRI data were obtained from 1365 women (aged 71–89) in the Women's Health Initiative Memory Study and local brain volumes were estimated using RAVENS (regional analysis of volumes in normalized space). Based on geocoded residential locations and air monitoring data from the U.S. Environmental Protection Agency, we employed a spatiotemporal model to estimate long-term (3-year average) exposure to ambient PM_2.5_ preceding MRI scans. Voxel-wise linear regression models were fit separately to gray matter (GM) and white matter (WM) maps to analyze associations between brain structure and PM_2.5_ exposure, with adjustment for potential confounders.

**Results:** Increased PM_2.5_ exposure was associated with smaller volumes in both cortical GM and subcortical WM areas. For GM, associations were clustered in the bilateral superior, middle, and medial frontal gyri. For WM, the largest clusters were in the frontal lobe, with smaller clusters in the temporal, parietal, and occipital lobes. No statistically significant associations were observed between PM_2.5_ exposure and hippocampal volumes.

**Conclusions:** Long-term PM_2.5_ exposures may accelerate loss of both GM and WM in older women. While our previous work linked smaller WM volumes to PM_2.5_, this is the first neuroimaging study reporting associations between air pollution exposure and smaller volumes of cortical GM. Our data support the hypothesized synaptic neurotoxicity of airborne particles.

## Introduction

Growing evidence suggests that exposure to ambient air pollutants, especially particulate matter (PM), is a novel environmental risk factor of brain aging (Block et al., 2012). Cross-sectional studies have indicated that residing in places with higher levels of fine particulate matter (i.e., PM_2.5_) is associated with poorer cognitive functioning in older adults (Ailshire and Crimmins, 2014; Gatto et al., 2014). Further support comes from longitudinal studies showing that greater ambient PM_2.5_ exposure is associated with accelerated cognitive aging (Weuve et al., 2012; Tonne et al., 2014). In addition, neurotoxic effects of exposure to particulate air pollutants on the brain have been reported in animal models (Fonken et al., 2011; Davis et al., 2013).

Despite increasing epidemiologic evidence linking late-life exposure to ambient air pollution with accelerated cognitive aging (Block et al., 2012), only a few studies have examined associations with brain structure in humans using neuroimaging data. Wilker et al. recently reported that greater residential exposure to PM_2.5_ was associated with smaller cerebral volumes in the Framingham Offspring Study (Wilker et al., 2015). We recently reported that participants in the Women's Health Initiative Memory Study (WHIMS) who lived for at least 6–7 years in places with greater levels of PM_2.5_ had smaller overall brain and white matter (WM) volumes compared to women with less exposure (Chen et al., 2015).

Both of the aforementioned studies used ROI-based analyses, which aggregate volumetric measures within pre-defined neuroanatomical regions and assume homogenous associations across all voxels within each ROI. While ROI-based analyses reduce the dimensionality of imaging data, regions of interest have to be defined in advance and the quality of the analyses depends on the precision of the segmentation approaches (Lee et al., 2015). Detecting patterns that extend continuously across multiple regions may be challenging for these approaches.

Voxel-based morphometry (VBM) is a complementary technique that measures local brain volumes in a normalized space and thus does not suffer from these limitations (Goldszal et al., 1998; Good et al., 2001). Our analyses are based on the Regional Analysis of Volumes Examined in Normalized Space (RAVENS) which is a well-validated form of voxel-based morphometry that preserves local tissue volumes after transformation to stereotaxic space (Davatzikos et al., 2001). The RAVENS approach has been extensively used in the last 15 years in large-scale neuroimaging studies such as Alzheimer's Disease Neuroimaging Initiative (Misra et al., 2009), Baltimore Longitudinal Aging Study (Davatzikos et al., 2009; Driscoll et al., 2012), WHIMS-MRI (Zhang et al., 2016), etc. We hypothesized that conducting more detailed analyses of the associations between air pollution neurotoxicity and local brain structure using RAVENS approaches would generate further insights about the impact of air pollution on brain structure.

## Methods

### Participants

The Women's Health Initiative Memory Study (WHIMS) investigated the effects of postmenopausal hormone therapy on the risk of dementia and changes in cognitive function in women aged 65–80 at enrollment (1996–1998) into the WHI randomized placebo-controlled clinical trials (Shumaker et al., 1998; Espeland et al., 2004). The WHIMS Magnetic Resonance Imaging study (WHIMS-MRI) study enrolled WHIMS participants from 14 of 39 sites, (Jaramillo et al., 2007; Resnick et al., 2009) from January 2005 through April 2006. Here we analyzed images from 1365 participants who met WHIMS-MRI reading criteria. These criteria were described previously (Coker et al., 2014). This study was also conducted in accordance with the Declaration of Helsinki. All participants provided written informed consent. This research was approved by the Wake Forest School of Medicine IRB.

### Image acquisition and pre-processing

MRI scans were performed using a standardized protocol developed by the MRI Quality Control Center in the Department of Radiology of the University of Pennsylvania. Details on procedures for acquisition and processing were published previously (Coker et al., 2009; Resnick et al., 2009). Briefly, the scans were obtained with a field of view = 22 cm and a matrix of 256 × 256. Included were oblique axial spin density/T2-weighted spin echo (TR:3200 ms, TE = 30/120 ms, slice thickness = 3 mm), fluid-attenuated inversion recovery (FLAIR) T2-weighted spin echo (TR = 8000 ms, TI = 2000 ms, TE = 100 ms, slice thickness = 3 mm), and oblique axial three-dimensional T1-weighted gradient echo (flip angle = 30 degrees, TR = 21 ms, TE = 8 ms, slice thickness = 1.5 mm) images from the vertex to the skull base parallel to the anterior commissure–posterior commissure (AC-PC) plane.

For voxel-based analyses, the T1-weighted images were preprocessed using the following steps: (1) alignment of the brain with the AC-PC plane; (2) removal of extracranial material; (3) tissue segmentation into gray matter (GM), white matter (WM), and cerebrospinal fluid (CSF), using a method described elsewhere (Zhang et al., 2016); (4) high-dimensional image warping to a standard MNI space through an elastic registration method (Shen and Davatzikos, 2002); (5) applying the deformation field that resulted from the spatial registration to the segmented images, thereby generating mass-preserved volumetric maps (or tissue density maps), named Regional Analysis of Volumes Examined in Normalized Space (RAVENS) maps (Davatzikos et al., 2001); (6) the RAVENS maps are normalized by the intracranial volumes to control for inter-subject differences in head size; (7) resampling the RAVENS maps to have 2 × 2 × 2 mm voxel size; and (8) smoothing of the GM and WM RAVENS maps using an 8 mm isotropic Gaussian kernel.

### Ambient air pollution data

We estimated residential exposures to PM_2.5_ from ambient sources, using a Bayesian Maximum Entropy (BME)-based spatiotemporal modeling approach. BME is a powerful stochastic modeling and mapping method for characterizing environmental exposure and human-ecosystem interactions (Christakos et al., 2001), which has been used in several large epidemiological cohort studies (Jerrett et al., 2013; Chen et al., 2015). In order to minimize the scaling error resulting from temporal misalignment in both the exposure source data and the subsequent estimates, a BME spatiotemporal model was constructed to produce daily ambient PM_2.5_ concentration at each geocoded location where WHIMS participants resided. To evaluate the validity of resulting exposure estimates, we conducted cross-validation analyses on the estimation accuracy, using US Environmental Protection Agency (EPA) air monitoring data. We first randomly divided the data into 10 distinctive sets of monitoring stations. For each “held-out” 10% of these data, we obtained daily BME estimates using only data from the remaining 90% of monitoring stations. We then pooled the cross-validation statistics across 10 distinctive sets and found moderate correlations between the “held-out” data and their BME estimates (cross-validation *R*^2^ = 0.74 for daily PM_2.5_). These daily BME estimates were then aggregated and combined with the residential histories, including relocations to calculate the 3-year average exposures preceding each brain MRI scan. These 3-year average exposures were highly correlated (Pearson's *R* = 0.93) with the cumulative exposure estimates of yearly PM_2.5_ used in previous work (Chen et al., 2015).

### Measurement of covariates

At the WHIMS enrollment, participants completed structured questionnaires to provide information on demographics (age, race/ethnicity), socioeconomic status (including education, family income, employment status), lifestyle factors (smoking, alcohol consumption), clinical characteristics (cardiovascular disease [CVD] and related risk factors), and prior hormone therapy use. History of CVD included previous coronary heart disease (myocardial infarction, coronary angioplasty, or coronary artery bypass graft), stroke, or transient ischemic attack. Body mass index (kg/m^2^) was calculated. Hypertension was defined as use of antihypertensive medication or elevated blood pressure (systolic blood pressure ≥140 mmHg or diastolic blood pressure ≥90 mmHg). Treated diabetes mellitus (DM) was defined as a physician diagnosis plus oral medications or insulin therapy. Good reliability and validity of both the self-reported medical histories and the physical measures have been documented (Heckbert et al., 2004).

### Statistical analysis

Voxel-wise linear regression models (Good et al., 2001) were fit to GM and WM RAVENS maps using Statistical Parametric Mapping (SPM) software (version 8) to examine the associations that brain structures had with PM_2.5_ exposure after adjusting for intracranial volume and potential confounders including age, race, BMI, geographic region (Northeast, South, Midwest, and West; Chen et al., 2015), education, family income, employment status, smoking, alcohol consumption, CVD history, hypertension, treated diabetes, and prior hormone therapy use. We investigated both negative and positive associations of PM_2.5_ with tissue volumes. All results were corrected for multiple comparisons using a false discovery rate (FDR) < 0.05 (Benjamini and Hochberg, 1995). Clusters with fewer than 50 voxels were removed from the results.

## Results

**Figure 1:**
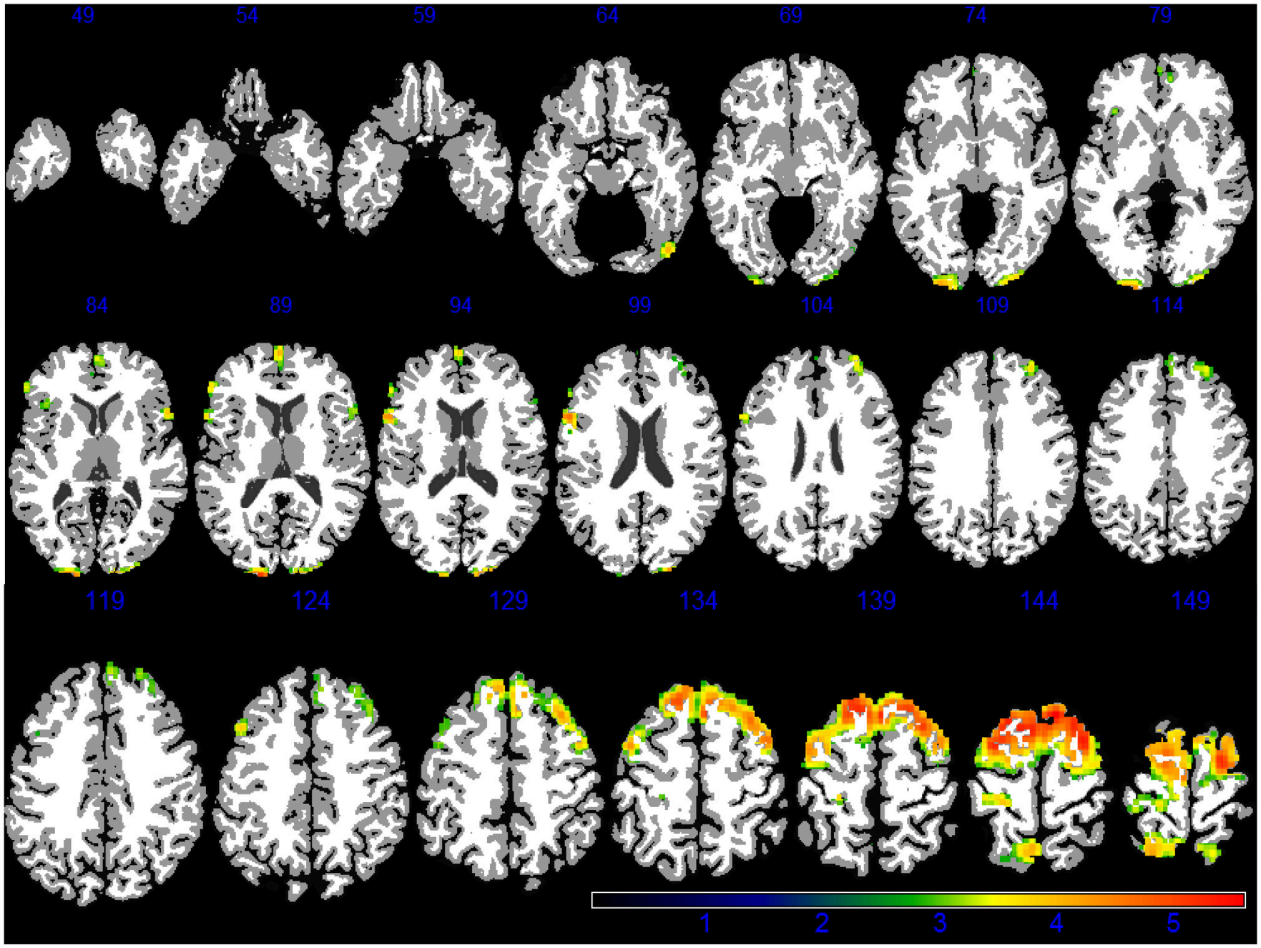
**GM areas negatively associated to PM_2.5_ exposure (*q* < 0.05 FDR corrected) in the VBM linear regression models are presented in color**. Images are oriented according to the neurological convention.

**Figure 2:**
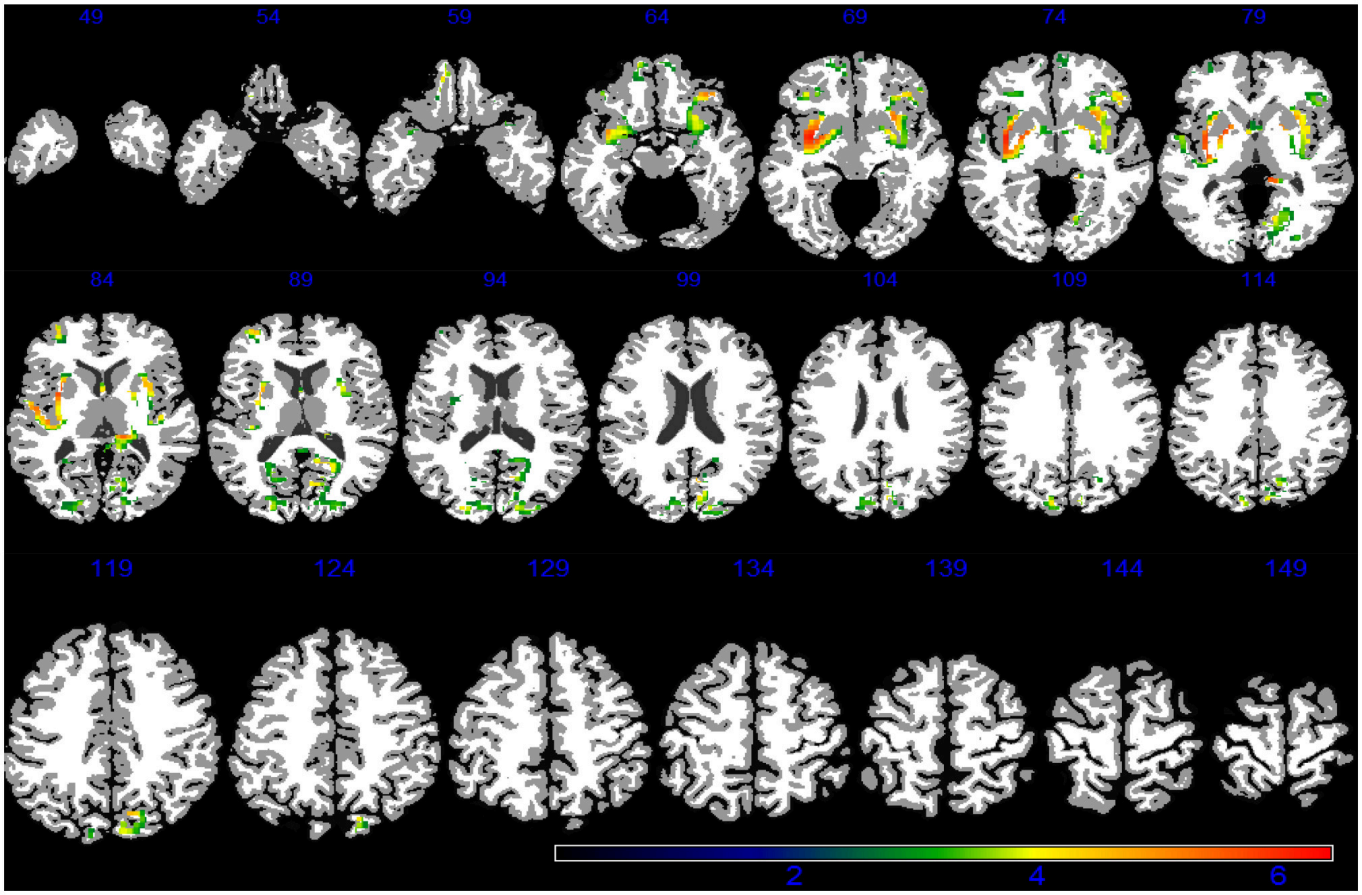
**WM areas with decreased volumes associated to increased PM_2.5_ exposure (*q* < 0.05 FDR corrected) in the VBM linear regression models are presented in color**. Images are oriented according to the neurological convention.

**Figure 3:**
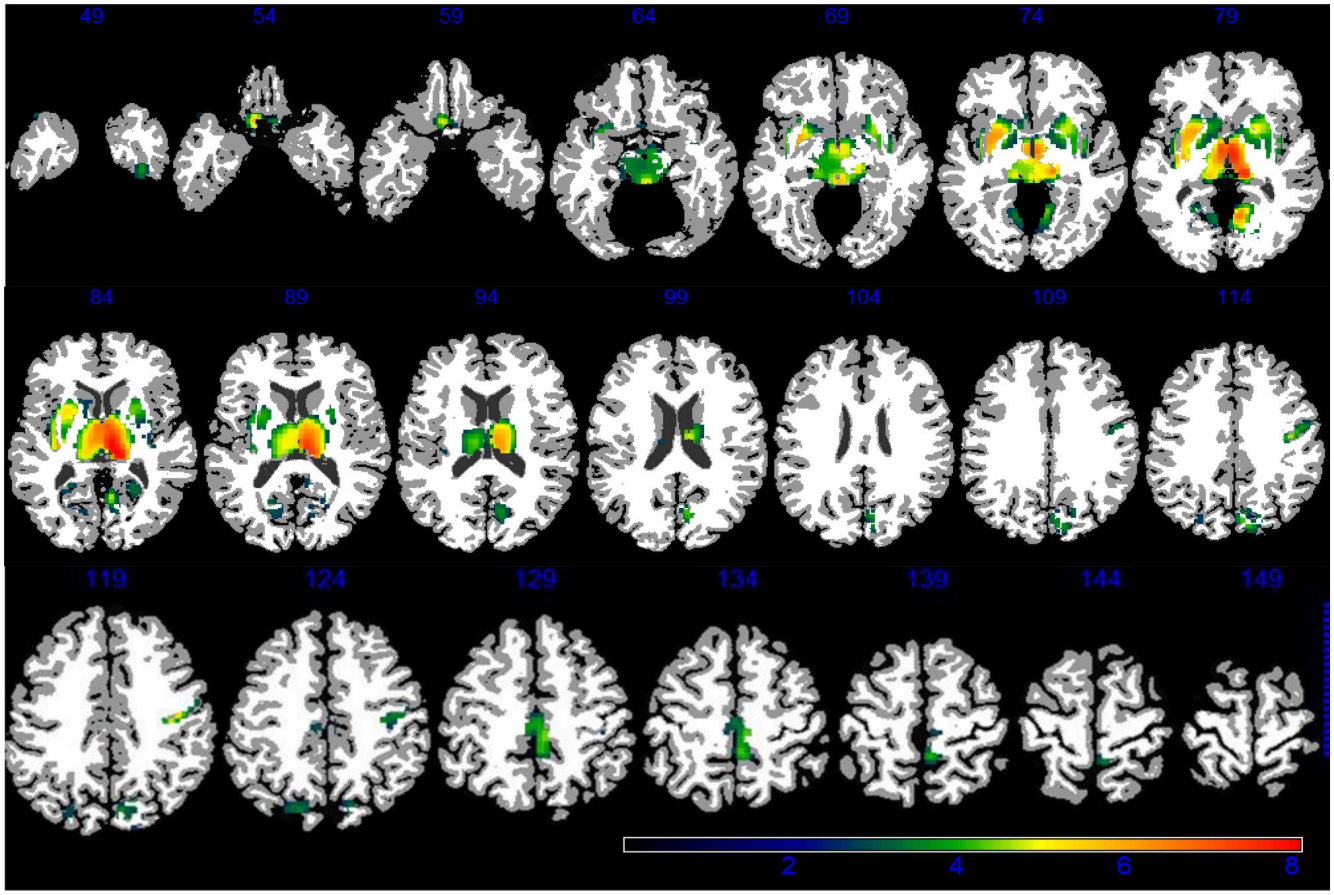
**GM areas positively associated to PM_2.5_ (*q* < 0.05 FDR corrected) according to VBM linear regression are presented in color**. Images are oriented according to the neurological convention.

Demographic, lifestyle, and clinical characteristics of participants are listed in Table 1. Greater PM_2.5_ exposure was associated with spatial patterns of smaller brain volumes in cortical GM and subcortical WM areas (Figures 1, 2). For GM, higher PM_2.5_ was associated with smaller volumes clustered in the bilateral superior, middle, and medial frontal gyri. Other clusters of negative associations were in the left inferior frontal gyrus and bilateral superior parietal lobule and occipital poles. For WM, the largest clusters of negative associations were in the anterior and posterior extreme/external capsule and the calcarine gyri. No correlation was found between corpus callosum and PM_2.5_ exposure. In addition, we found no evidence for smaller hippocampal or temporal lobe volumes with PM_2.5_ exposure. Statistically significant clusters of associations were found in deep gray matter nuclei (*q* < 0.05 FDR corrected): larger volumes were associated with increased PM_2.5_ exposures (Figure 3). These local GM regions, pinpointed by colors in Figure 3, included the thalamus, putamen, and globus pallidus bilaterally, as well as the posterior insula. There were no WM areas with significant positive associations with increased PM_2.5_ exposure.

**Table 1:** **Baseline characteristics of WHIMS-MRI participants (*N* = 1365)**.

**Variable**	**Mean (*SD*) Frequency (%)**
Age	70.53 (3.64)
Body mass index	28.23 (5.43)
**Race/Ethnicity**	
Black/African–American	61 (4.47%)
Hispanic/Latino	19 (1.39%)
White	1245 (91.21%)
Other	40 (2.93%)
**Education**	
<High school	60 (4.41%)
High school/general education degree	317 (23.27%)
>High school	985 (72.32%)
**Employment**	
Currently employed	246 (18.05%)
Not working	142 (10.42%)
Retired	975 (71.53%)
**Region**	
Northeast	310 (22.71%)
South	204 (14.95%)
Midwest	477 (34.95%)
West	374 (27.40%)
**Smoking**	
Never	784 (57.90%)
Past	513 (37.89%)
Current	57 (4.21%)
**Alcohol**	
Non-drinker	176 (13.00%)
Past drinker	225 (16.62%)
<1 drink per day	800 (59.08%)
>1 drink per day	153 (11.30%)
High cholesterol requiring pharmaceutical treatment	215 (16.06%)
Cardiovascular disease ever	187 (13.87%)
Hypertension ever	491 (36.21%)
Prior use of hormone therapy	6733 (46.37%)
Diabetes treated ever (oral therapy or injected insulin)	44 (3.23%)
Baseline score on mini-mental examination	96.10 (3.45)

## Discussion

Our detailed analyses identified specific subcortical areas in which smaller WM volumes were associated with greater PM_2.5_ exposure, namely the external and extreme capsule and the calcarine cortices. This observation suggests that regions involved in important functional networks, such as the salience and visual networks, appear to be affected by ambient PM_2.5_. We also found that ambient PM_2.5_ exposure was associated with local GM brain structures. Our findings provide the first epidemiologic evidence that PM_2.5_-induced neurotoxic effects may involve structural damage to cortical GM. In cohorts like WHIMS-MRI participants, lower GM volumes may reflect shrinkage of neurons, reductions of synaptic spines, and dendritic arborization, and lower numbers of synapses (Fjell and Walhovd, 2010) rather than neuronal loss. To date, there is limited data from animal studies showing evidence for PM-induced neuronal toxicity, including the reduction of dopaminergic neurons in the striatum of genetically-modified mice (Veronesi et al., 2005) exposed to concentrated PM_2.5_ representing the ambient background and cortical neuronal loss in rats with *oral* ingestion of PM from vehicular emissions with unspecified particle sizes (Ejaz et al., 2014). However, there is growing evidence that synaptic neurotoxicity results from exposure to ambient particles. In the mouse hippocampus, impaired synaptic function is induced by short-term *in vitro* exposure to particulate matter from urban traffic (Davis et al., 2013). Reduced synaptic plasticity (decreased dendritic spine density and branching) may result from long-term inhaled exposure to ambient PM_2.5_ (Fonken et al., 2011).

The associations between PM_2.5_ exposure and patterns of smaller GM volumes that we identified were primarily in the dorsolateral and medial prefrontal cortex, regions associated with higher cognitive function such as working memory, episodic memory retrieval, and executive function. Age-related deficits in retrieval of episodic memory have been associated with volume reductions and functional changes in the middle frontal gyrus (Buckner et al., 2000; Raz et al., 2005). Weuve et al. reported memory function declined in older women (70–81 years) living in locations with higher PM_2.5_ exposures (Weuve et al., 2012). Two other studies also reported associations between PM_2.5_ exposure and low performance of episodic memory (Ailshire and Crimmins, 2014; Tonne et al., 2014).

We found little evidence that PM_2.5_ exposure was related to hippocampal volume. This is consistent with two previous studies employing ROI-based analyses (Chen et al., 2015; Wilker et al., 2015). This null finding may be influenced by the nature of the cohort and characteristics of the exposure. Longitudinal brain MRI studies have shown that loss of hippocampal volume starts in young adulthood, with age-related accelerated shrinkage in the mid-50s (Raz et al., 2005). Long-term (10-month) exposure to concentrated ambient PM_2.5_ decreased dendritic spine density in hippocampal CA1 neurons of 4-week-old wild-type mice (C57BL/6; Fonken et al., 2011). It is therefore possible that PM_2.5_ exposure affects hippocampal volume in early- or mid-life. Also, because our exposure estimation relied exclusively on EPA's ambient monitoring data, we cannot exclude the possibility that reduced hippocampal volume might be found in older adults exposed to other particulate matter with different profiles of neurotoxicity (e.g., the ultrafine particles from vehicular exhausts; Davis et al., 2013).

The positive associations we observed between PM_2.5_ exposure and GM volumes in basal ganglia were unexpected, and the potential underlying mechanisms are unclear. In our previous ROI-based analyses, we found no positive associations between PM_2.5_ exposure and basal ganglia volume (Chen et al., 2015). Experimentally, exposure to small particles may result in a loss of dopaminergic neurons in striatum, as shown with *in vitro* (Gillespie et al., 2013) or inhalation exposure (Veronesi et al., 2005) to concentrated ambient particles. These results would predict an association between PM_2.5_ and smaller volumes of basal ganglia. On the other hand, environmental exposures to paramagnetic substances (e.g., magnesium and iron) may distort T1-weighted images and interfere with volumetric estimation (Goto et al., 2013; Lorio et al., 2014). One recent neuropathological study identified (Maher et al., 2016) the magnetite nanoparticle from environmental sources in human brains, and others have shown that airborne particles with magnetic properties are abundant in polluted cities (Gargiulo et al., 2016). Larger basal ganglia volumes have been linked to some pathological processes in the brain (e.g., schizophrenia; Mamah et al., 2007) and use of antipsychotic medications(Scherk and Falkai, 2006). However, we are unaware of prior studies showing increased psychiatric disease/use of antipsychotics or changes in paramagnetic properties resulting from long-term PM_2.5_ exposure. It is also unclear why higher PM_2.5_ exposure would be associated with larger volumes in the thalamus and lenticular nucleus, but have a less pronounced effect on caudate. Our sample size was unusually large for VBM analyses, which could mean that we detected subtle differences not readily identified in previous VBM studies.

Our findings strengthen the evidence that WM architecture may represent a novel target of particle-induced neurotoxicity. While associations with GM volumes are largely restricted to frontal gyri (Figure 1), the impact of PM_2.5_ on WM volumes appears to be more regionally-distributed (Figure 2) and involves the same regions (frontal, parietal, and temporal lobes) as previously reported in our ROI-based study (Chen et al., 2015). These observed differences in affected brain regions raise the interesting possibility that the smaller WM volumes reflect adverse effects on oligodendrocytes and/or myelin damage, while smaller GM volumes may imply synaptic neurotoxicity, both possibly resulting from long-term PM_2.5_ exposure. Investigation on the neurobiological mechanisms (e.g., neuroinflammation, oxidative stress) linking PM exposure to central neurotoxicity is an active area of research in environmental neurosciences, likely involving multi-level pathways perturbed at the molecular levels (e.g., activation of TNF-alpha; Levesque et al., 2011; Cheng et al., 2016), selected target tissues (e.g., remodeling glutamatergic synapses; Morgan et al., 2011), and interactions among different neural cells (e.g., neuron-glial interaction; Block and Calderón-Garcidueñas, 2009) and across systems (e.g., via the neurohormonal stress response to air pollution; Kodavanti, 2016). Subclinical cerebrovascular injuries may also result in loss of brain tissues, although published neuroimaging studies with late-life exposure to PM_2.5_ so far (Chen et al., 2015; Wilker et al., 2015, 2016) had not produced strong evidence for this neurovascular pathway linking air pollution to brain aging.

One recent cross-sectional study also showed that early-life PM_2.5_ exposure may affect age-related WM maturation (Peterson et al., 2015). In a sample of 40 minority urban-dwelling school-age children, prenatal exposures to polycyclic aromatic hydrocarbons (measured from personal air samples of PM_2.5_ during pregnancy) was associated with a smaller local WM volume, as indicated by the reduction of surface areas (Peterson et al., 2015). PM-induced WM damage, as reflected by hypomyelination and aberrant white matter structural integrity were recently demonstrated in mouse models with early-life exposure to concentrated ambient ultrafine particles (Allen et al., 2015). Beyond volumetric measures, future studies should consider diffusion tensor tractography (Madden et al., 2009) and MR spectroscopy (Bray and Mullins, 2014) to better understand the WM connectomes and molecular profilles potentially disrupted by PM exposure. To elucidate the neuropathology and mechanisms underlying the observed neurotoxicity on WM, we also need to understand whether PM_2.5_ exposure results in myelination disturbance (Kohama et al., 2012) and age-related decrease of the oligodendrocytes in subcortical WM (Chen et al., 2011). VBM and ROI methods operate based on different assumptions. Thus, while often they show some degree of coincidence, they also can lead to different findings. It is interesting to note that the present VBM analyses did not reveal a statistically significant association between PM_2.5_ and corpus callosum, in contrast to our findings using ROI based methods (Chen et al., 2015). In one study of schizophrenic patients (Giuliani et al., 2005), despite some similarities in results, there also were brain areas uncovered differentially by each of the methods. In general, VBM and ROI approaches are complementary; their relative effectiveness is likely related to the specific shape of the spatial patterns of brain tissue atrophy and the image warping and segmentation methods used to preprocess the MRI data. Finally, we used in our analyses RAVENS maps that were ICV adjusted as part of the image preprocessing. The ICV-adjustment strategies (e.g., proportional, residuals, nuisance covariate, etc.) have been the subject of debate in the past (Arndt et al., 1991; Barnes et al., 2010) and more recently (Voevodskaya et al., 2014; Nordenskjöld et al., 2015). While other analyses are possible, they would be beyond the scope of this particular paper.

Our study has some limitations. First, our analyses were based on cross-sectional measures of brain volume. Longitudinal studies with repeated brain MRI scans are needed to characterize associations with rates of changes in brain volumes. We only studied older women, so our findings may not generalize to men. This cohort was composed of relatively well-educated and mostly Caucasian women, which may not be representative of the general population. We only studied PM_2.5_, and have not assessed emission sources, particle constituents, or interactions with other pollutant mixtures. The lack of nationwide monitoring data before 1999 prevented us from assessing the impact of earlier exposures. Finally, long-term chronic exposure, especially if accumulated since mid- or earlier life, might have different—and potentially greater—adverse effects than what we observed.

## Conclusions

This first neuroepidemiologic VBM analysis of brain regions associated with air pollution provides further evidence for the adverse effect of particulate air pollutants on brain structure in older women. Long-term PM_2.5_ exposures are linked to potential loss of brain volume in both GM and WM tissues, but in different brain networks. Longitudinal studies are needed to clarify the sequence of pathogenetic events associated with long term exposure to fine particles.

## Investigators participating in the WHIMS-MRI

**WHIMS-MRI Clinical Centers:** Albert Einstein College of Medicine, Bronx, NY: Sylvia Wassertheil-Smoller, Mimi Goodwin, Richard DeNise, Michael Lipton, James Hannigan, Anthony Carpini, David Noble, Wilton Guzman; Medical College of Wisconsin, Milwaukee: Jane Morley Kotchen, Joseph Goveas, Diana Kerwin, John Ulmer, Steve Censky, Troy Flinton, Tracy Matusewic, Robert Prost; Stanford Center for Research in Disease Prevention, Stanford University, CA: Marcia L. Stefanick, Sue Swope, Anne Marie Sawyer-Glover, Susan Hartley; The Ohio State University, Columbus: Rebecca Jackson, Rose Hallarn, Bonnie Kennedy, Jill Bolognone, Lindsay Casimir, Amanda Kochis; University of California at Davis, Sacramento: John Robbins, Sophia Zaragoza, Cameron Carter, John Ryan, Denise Macias, Jerry Sonico; University of California at Los Angeles: Lauren Nathan, Barbara Voigt, Pablo Villablanca, Glen Nyborg, Sergio Godinez, Adele Perrymann; University of Florida, Gainesville/Jacksonville: Marian Limacher, Sheila Anderson, Mary Ellen Toombs, Jeffrey Bennett, Kevin Jones, Sandy Brum, Shane Chatfield, Kevin Vantrees; University of Iowa, Davenport: Jennifer Robinson, Candy Wilson, Kevin Koch, Suzette Hart, Jennifer Carroll, Mary Cherrico; University of Massachusetts, Worcester: Judith Ockene, Linda Churchill, Douglas Fellows, Anthony Serio, Sharon Jackson, Deidre Spavich; University of Minnesota, Minneapolis: Karen Margolis, Cindy Bjerk, Chip Truwitt, Margaret Peitso, Alexa Camcrena, Richard Grim, Julie Levin, Mary Perron; University of Nevada, Reno: Robert Brunner, Ross Golding, Leslie Pansky, Sandie Arguello, Jane Hammons, Nikki Peterson; University of North Carolina, Chapel Hill: Carol Murphy, Maggie Morgan, Mauricio Castillo, Thomas Beckman, Benjamin Huang; University of Pittsburgh, PA: Lewis Kuller, Pat McHugh, Carolyn Meltzer, Denise Davis, Joyce Davis, Piera Kost, Kim Lucas, Tom Potter, Lee Tarr.

**WHIMS-MRI Clinical Coordinating Center:** Wake Forest School of Medicine, Winston-Salem, NC: Sally Shumaker, Mark Espeland, Laura Coker, Jeff Williamson, Debbie Felton, LeeAnn Gleiser, Steve Rapp, Claudine Legault, Maggie Dailey, Ramon Casanova, Julia Robertson, Patricia Hogan, Sarah Gaussoin, Pam Nance, Cheryl Summerville, Ricardo Peral, Josh Tan.

**WHIMS-MRI Quality Control Center:** University of Pennsylvania, Philadelphia: Nick Bryan, Christos Davatzikos, Lisa Desiderio.

**U.S. National Institutes of Health:** National Institute on Aging, Bethesda, MD: Neil Buckholtz, Susan Molchan, Susan Resnick; National Heart, Lung, and Blood Institute, Bethesda, MD, Jacques Rossouw, Linda Pottern.

## Author contributions

Design/Conceptualization of the study (RC, ME, JC); Acquisition of data (RC, ME, JC, MS, WV, JR, YA); Analysis of the data (RC, XW, ME, JC, MS, WV, HC, SR); Interpretation of the data (RC, ME, JC, HC, SR); Drafting the manuscript (RC, ME, JC); Critical revision of the manuscript for important intellectual content (All listed authors); Final approval of the current version of this manuscript (All listed authors).

## Funding

The Women's Health Initiative is funded by the National Heart, Lung, and Blood Institute. The Women's Health Initiative Memory Study was funded in part by Wyeth Pharmaceuticals, Inc., St. Davids, PA. The research work was supported by R21AG051113-01 (PIs: RC and JC) R01AG033078 (PI: JC). HC is supported by P50-AG05142. SR is supported by the Intramural Research Program, NIA, NIH.

### Conflict of interest statement

ME has served on an advisory panel for Takeda Global Research and Development. He currently serves on a steering committee for Boehringer-Ingelheim Pharmaceuticals. He serves on the editorial board for the Journal of Gerontology Medical Sciences. The other authors declare that the research was conducted in the absence of any commercial or financial relationships that could be construed as a potential conflict of interest.
